# Brain Gene Expression in a Novel Mouse Model of Postpartum Mood Disorder

**DOI:** 10.1515/tnsci-2019-0030

**Published:** 2019-08-07

**Authors:** Trevor Humby, William Davies

**Affiliations:** 1 School of Psychology, Cardiff University, Cardiff CF10 3AT, Cardiff, UK; 2 Neuroscience and Mental Health Research Institute, Cardiff University, Cardiff CF24 4HQ, UK; 3 Medical Research Council Centre for Neuropsychiatric Genetics and Genomics and Division of Psychological Medicine and Clinical Neurosciences, School of Medicine, Cardiff University, Cardiff CF24 4HQ, UK

**Keywords:** Female, G-protein coupled receptor, mice, postpartum depression, postpartum psychosis, steroid sulfatase

## Abstract

**Background:**

Steroid sulfatase (STS) cleaves sulfate groups from steroid hormones; its expression/activity increases in late pregnancy and into the postpartum period. STS-deficient human and mouse mothers display elevated psychopathology and abnormal behaviour respectively; in mice, these effects can be partially normalised by antipsychotic (ziprasidone) administration.

**Methodology:**

We compared brain gene expression in new mouse mothers administered the STS inhibitor 667-Coumate, or vehicle; significant changes were followed-up with pathway analysis and quantitative polymerase chain reaction (qPCR). Finally, the effects of combined 667-Coumate and ziprasidone administration on expression of the most robustly differentially-expressed genes were examined.

**Results:**

Surprisingly, no between-group gene expression changes were detected at a False Discovery Rate (FDR)-corrected p<0.1. 1,081 unique expression changes were detected at p<0.05, two top hits were verified by qPCR, and pathway analysis indicated enrichment of genes involved in olfactory transduction. The expression of Stoml3 and Cyp2g1 was unaffected by ziprasidone administration.

**Conclusions:**

Postpartum behavioural abnormalities in STS-deficient mothers are likely to be the culmination of many small gene expression changes. Our data are consistent with the idea that olfactory function is key to maternal behaviour in mice, and suggest that aberrant expression of olfactory system genes may underlie abnormal maternal behaviour in STS-deficient women.

## Introduction

Steroid sulfatase is an enzyme which cleaves sulfate groups from a variety of steroid hormones e.g. dehydroepiandrosterone sulfate (DHEAS), thereby altering their solubility and activity [1]. STS is expressed in numerous mammalian tissues, with highest expression in the placenta (www.ncbi.nlm.nih.gov/unigene/); in the developing and adult human brain, relatively high STS expression and activity is seen in the cortex, thalamus, cerebellum, basal ganglia, hippocampus and hypothalamus [2,3]. STS deficiency is associated with increased developmental and mood disorder risk and a number of behavioural differences including: inattention, increased impulsivity and altered mood and social function [4, 5, 6]; these behavioural differences may be mediated, in part, by underlying changes in serotonergic or cholinergic function [7, 8, 9]. Depression, prior and current history of medical disorders, and cognitive impairment, have previously been highlighted as important risk factors in suicidality [10].

In both humans and rodents, STS expression and activity increases in brain and peripheral tissues towards the end of pregnancy and into the postpartum period; hence, enzyme deficiency or dysfunction could potentially be associated with postpartum psychopathology [11,12]. Consistent with this, we have recently shown that women who are heterozygous for genetic mutations encompassing *STS* are at increased risk of postpartum mood disorders [4]. We have also demonstrated that female mice, in which STS activity is acutely, and systemically, inhibited with 667-Coumate shortly after giving birth, show altered maternal behaviour (specifically anxiety-related and startle phenotypes) relative to vehicle-treated mice; these drug-induced behavioural abnormalities can be partially reversed by concurrent administration of the atypical antipsychotic drug ziprasidone [13].

To investigate the neurobiology underlying the postpartum behavioural phenotypes in STS deficient individuals, we compared whole brain gene expression in behaviourally-defined 667-Coumate and vehicle-treated new mouse mothers. Given the large between-group behavioural differences, we suspected that screening by microarray would be able to readily identify robust gene expression differences and candidate biological pathways, and we reasoned that analysis of whole brain tissue would capture activity changes across multiple interacting brain regions (no single brain region has yet been especially strongly implicated in postpartum mood disorders). We successfully identified a number of nominally-significant gene expression differences, implicating a specific biological pathway.

## Methods

### Drug administration and behavioural analysis

Within 12hr of giving birth, female mice were injected with either 10mg/kg 667-Coumate or vehicle solution *per os* (n=12 per group); they were then injected with the same solution 48hr later, and behaviourally-tested 12hr after that. Coumate, and its relative 667-Coumate, provide highly potent and specific STS inhibition in rodents (>70% enzyme inhibition in liver and brain)

when administered in the aforementioned manner [14,15]. There is some evidence that 667-Coumate also binds carbonic anhydrase in erythrocytes, an observation which may explain its *in vivo* stability and deliverability [16]. Behavioural testing comprised of sequential assessment on the elevated plus maze, in a locomotor activity chamber, and in a startle/prepulse inhibition paradigm. A second batch of mice had 667-Coumate injections as described above, but also ziprasidone injections i.p. 24hr after giving birth, and 1hr prior to behavioural testing at doses of either 0, 0.3 or 1.0mg/kg (n=11, n=11 and n=7 respectively). Between-group behavioural measures were compared using parametric (two-tailed unpaired t-test or One Way ANOVA) or non-parametric (Mann Whitney U test or Kruskal Wallis test) statistics depending upon normality of the data as determined by Shapiro-Wilk test. Results are presented as either mean±standard deviation of the mean or median with 95% confidence intervals determined by bootstrapping for normal and non-normal data respectively. Experiments were performed according to the UK Animal Scientific Procedures Act (1986).

### Tissue collection, RNA extraction and cDNA synthesis

3hr after behavioural testing, subjects were culled by cervical dislocation; whole brains were immediately removed, bisected sagitally, and frozen on dry ice. The 3hr post-behaviour timepoint was chosen in order to allow acute physiological changes induced by moderately aversive behavioural experiences to return to baseline. High-quality total RNA was extracted from the right hemisphere of the brain using RNeasy Plus Universal Midi Kit (Qiagen) according to the manufacturer’s instructions. For microarray analysis, three biological replicates per group were generated, with each replicate containing equal amounts of RNA pooled from four hemibrains; 260/280 absorbance ratios of 2.05-2.08 and RIN numbers of 9.4-9.8 were recorded for the six replicates using Agilent 2100 Bioanalyzer (Agilent Technologies, Palo Alto, CA, USA). 20μl cDNA solution per hemibrain was synthesised from 4-5 μg RNA using RNA-to-cDNA EcoDry Premix with random primers (Clontech), and was diluted 50-fold with distilled water.

### Microarray hybridisation and bioinformatic analysis

Microarray analysis was conducted by Central Biotechnology Services at Cardiff University according to standard protocols. Briefly, biotin-labelled targets for the microarray experiment were prepared using 100ng of total RNA per replicate. Sense single-stranded cDNA was synthesized, fragmented and labelled using the Genechip WT PLUS Reagent Kit (Affymetrix) in conjunction with the Affymetrix Genechip Poly-A RNA Control Kit as described in the User Manual (P/N 703174). A hybridization cocktail containing the biotinylated target was incubated with the GeneChip Mouse 2.0 ST array (Affymetrix) at 60rpm for 16hr at 45°C in a Genechip Hybridisation Oven 645. After hybridization, non-specifically bound material was removed by washing, and specifically-bound target was detected using the GeneChip Hybridization, Wash and Stain Kit, in conjunction with the GeneChip Fluidics Station 450 (Affymetrix). The arrays were scanned using a GeneChip Scanner 3000 7G (Affymetrix) in conjunction with Affymetrix Genechip Command Console (AGCC) software. Data were normalised using Robust Multiarray Average (RMA); the overall pattern of normalised expression data appeared equivalent across the six replicates. Differential gene expression analysis was performed using lmFit and eBayes from the Limma package [17]. FDR-corrected and nominal p-values were generated for each probe, with values<0.05 being regarded as nominally statistically-significant. Pathway analysis was performed using Database for Annotation, Visualisation and Integrated Discovery version 6.8 (DAVID, RRID: SCR_001881) [18]. Raw microarray data are available in the ArrayExpress database (http://www.ebi.ac.uk/arrayexpress RRID: SCR_002964) under accession number E-MTAB-7233.

### Quantitative Polymerase Chain Reaction (qPCR)

qPCR was performed using a Rotorgene 6000 coupled with a CAS1200 automated set-up, and utilizing standard consumables (Qiagen, Manchester, UK). PCR reactions were performed using 5μl cDNA mix and 200nM custom-designed primers (**Table 1**) and SensiMix (Bioline, London, UK). qPCR data were analysed using ΔC_t_ methods as described previously [19] with normalisation to the mean of three ‘housekeeping gene standards’ (*Hprt*, *Gapdh* and *Rn18s*) whose expression was significantly (p<0.001) correlated within the samples. Groups were compared by either Mann-Whitney U test, unpaired t-test or One Way ANOVA.

**Table 1:** Primer sequences used for quantitative PCR analysis. Primers were designed to allow optimum amplification, to span intron-exon boundaries where possible, and to amplify key coding gene transcripts.

Gene	Forward primer 5’-3’	Reverse primer 5’-3’
*Gapdh*	GAACATCATCCCTGCATCCA	CCAGTGAGCTTCCCGTTCA
*Hprt*	TTGCTCGAGATGTCATGAAGGA	AATGTAATCCAGCAGGTCAGCAA
*Rn18s*	GTAACCCGTTGAACCCCATT	CCATCCAATCGGTAGTAGCG
*Cyp2g1*	TGTCACACGGGACACTCATT	TGGGTAGCGGAAGTATTTGG
*Stoml3*	TGCAGCAGAGGGAGAAATGA	TCGGCAAGGGAAACACAATG
*Vmn1r33*	GTCATGCTGACCACAAGTGC	CAAGTGGCTGTCGCTATGAA
*Saa1*	GGTCTGGGCTTCTTCCTACC	TACCCTCTCCTCCTCAAGCA
*Orm3*	TCATCATGTTGAGCCTCCTG	GTCAGCCACAGCAATGAGAA
*Oxt*	CCATCACCTACAGCGGATCT	CACTTGCGCATATCCAGGTC

## Results

### Behavioural and endocrine comparison in animals used for microarray analysis

A subset of vehicle and 667-Coumate-treated mice from [13] which differed maximally on pertinent behavioural measures were selected for subsequent analysis, with the rationale being that these animals would exhibit the greatest difference in brain gene expression. 667-Coumate and vehicle-treated mothers differed significantly with respect to numbers of rears on the elevated plus maze (57 (95%CI:19-86.5) vs. 0 (95%CI:0-0), Mann-Whitney U=10.5, p<0.0001), latency to enter the open arm of the elevated plus maze (2.2(95%CI:2.0-3.0)s vs. 8.5s(95%CI:3.2-12.2), Mann-Whitney U=28.0, p=0.01), and startle response at 120dB covarying for bodyweight and baseline activity (51.6±8.7 vs. 62.5±8.3 abitrary units, t[1,20]=4.61, p=0.04). These group differences were not confounded by differential locomotor activity as indexed by infra-red beam breaks per hour (1118 (95%CI:925-2284) vs. 1600 (95%CI:786-2143), Mann-Whitney U=72.0, p=1.0), nor by number of live pups born to each mother (7 (95%CI:6-8) vs. 7(95%CI:6-8), Mann-Whitney U=71.0, p=0.95). The median serum DHEAS:DHEA ratio in 667-Coumate-treated mothers (n=11) was ~1.6-fold higher than that in vehicle-treated mothers (n=5)(333 vs. 211), indicating efficacy of systemic STS inhibition by the drug.

### Significantly-differentially expressed genes in the microarray analysis

We identified 1,081 unique differentially-expressed transcripts with a nominal p-value cut-off<0.05; the 24 most highly differentially expressed of these (fold change>1.5) are listed in **Table 2**. No significantly differentially expressed genes were identified using the comparatively stringent cut-off of False Discovery Rate (FDR)-corrected p-value of <0.1.

**Table 2:** Most highly-differentially expressed genes (>1.5 fold change, p<0.05) between vehicle and 667-Coumate-treated whole mouse brain according to microarray analysis

Gene	Gene product	Fold change	Human orthologue and genomic location
	*Expression upregulated in 667-Coumate-treated animals*		
*Cyp2g1*	Cytochrome P450, family 2, subfamily g, polypeptide 1	2.47	*CYP2G1P* (19q13.2)
*Ighg*	Immunoglobulin heavy chain (gamma polypeptide)	1.83	None
*Gm6890*	Unknown	1.71	*CTAG2* (Xq28)
*Stoml3*	Stomatin (Epb7.2)-like 3	1.66	*STOML3* (13q13.3)
*mir883a*	microRNA 883a	1.66	None
*Vmn1r33*	Vomeronasal 1 receptor 33	1.63	None
*4930529C04Rik*	Zinc finger, BED domain containing 4 pseudogene	1.63	None
*1700023F02Rik*	lincRNA	1.56	None
*Vmn2r14*	Vomeronasal 2, receptor 14	1.50	None
	*Expression downregulated in 667-Coumate-treated animals*		
*Saa1*	Serum amyloid A 1	1.76	*SAA1* (11p15.1)
*mir133a-2*	microRNA 133a-2	1.76	None
*Gsdmc3*	Gasdermin C3	1.72	*GSDMC3* (8q24.21)
*Klk1b22*	Kallikrein 1-related peptidase b22	1.66	*KLK1* (19q13.33)
*Orm3*	Orosomucoid 3	1.60	*ORM2* (9q32)
*Dynlt1b*	Dynein light chain Tctex-type 1B	1.58	*DYNLT1* (6q25.3)
*Olfr1454*	Olfactory receptor 1454	1.56	*OR5B3* (11q12)
*Svs3b*	Seminal vesicle secretory protein 3B	1.56	None
*AF357399*	snoRNA	1.56	None
*Olfr851*	Olfactory receptor 851	1.55	*OR7G2* (19p13.2)
*Olfr1039*	Olfactory receptor 1039	1.54	None
*Olfr1054*	Olfactory receptor 1054	1.54	*OR8K3* (11q12.1)
*Olfr911-ps1*	Olfactory receptor 911, pseudogene 1	1.54	None
*Aldoart1*	Aldolase 1 A, retrogene 1	1.53	*ALDOA* (16p11.2)
*Olfr1255*	Olfactory receptor 1255	1.52	*OR4C12* (11p11.12)

### Pathway analysis

Pathway analysis of the 1,081 differentially-expressed genes at p<0.05 with the mouse genome as background identified only one KEGG (Kyoto Encyclopedia of Genes and Genomes [20]) pathway, olfactory transduction, as being significant (Benjamini-corrected p=1.8x10^-3^). The following gene ontology (GO) terms for biological pathway were significant after correction for multiple testing (G-protein coupled receptor signalling p=1.2x10^-3^ and sensory perception of smell p=7.5x10^-4^), as were the following GO terms for molecular function (G-protein coupled receptor activity p=5.1x10^-5^ and olfactory receptor activity p=1.3x10^-4^). We tested the possibility that identification of the olfactory pathway could have been artefactual due to the relatively low and variable expression of the associated genes, by filtering for low expression variability within the following R Package using the nsFilter function with default value of var. cutoff 0.5: https://www.rdocumentation.org/packages/genefilter/versions/1.54.2/topics/nsFilter This analysis resulted in just 213 unique genes that were differentially-expressed at a nominal p-value<0.05, 35 of which had a fold change in expression >1.5 (the 24 genes from **Table 2** as well as *Psg25*, *1700047G07Rik*, *Ugt2b5*, *Vmn1r86*, *Olfr493*, *Olfr295*, *Vmn1r37*, *Olfr800* and *Fpr2*); for the 213 genes, the KEGG pathway ‘olfactory transduction’ remained highly significant (p=3.6x10^-11^), as did the GO biological pathway terms ‘G-protein-coupled receptor signalling’ and ‘sensory perception of smell’ (p=7.9x10^-12^ and p=1.2x10^-12^ respectively) and the GO molecular function terms ‘G-protein coupled receptor activity’ and ‘olfactory receptor activity’ (p=9.6x10^-17^ and 2.4x10^-13^ respectively).

### Quantitative PCR (qPCR)

**Figure 1:**
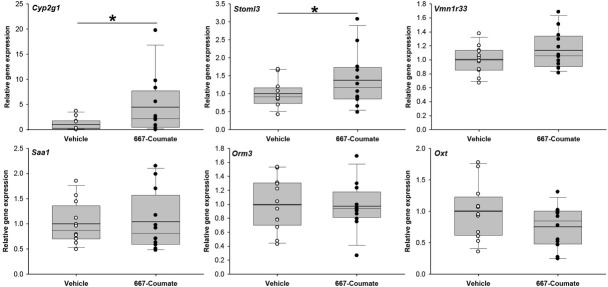
Comparison of gene expression in vehicle and 667-Coumate treated whole mouse brain by quantitative PCR. Boxes show interquartile range with median and mean (bold line) values, and whiskers represent 5% and 95% confidence intervals. *p<0.05.

**Figure 2:**
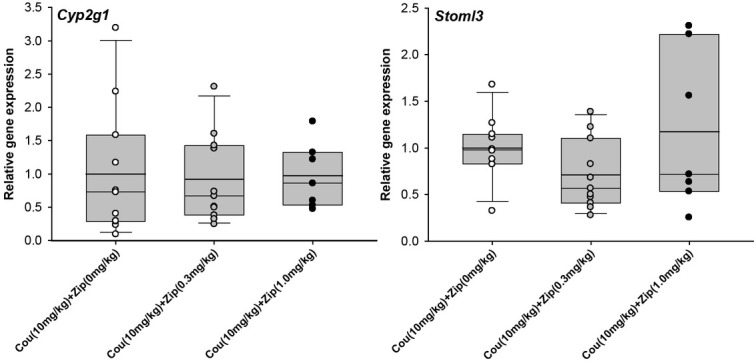
Comparison of gene expression in whole mouse brain by quantitative PCR in animals administered 667-Coumate and either 0, 0.3 or 1.0mg/kg ziprasidone. Boxes show interquartile range with median and mean (bold line) values, and whiskers represent 5% and 95% confidence intervals.

qPCR analysis was performed for five of the most highly-differentially expressed genes from **Table 2**, as well as for *Oxt* (downregulated 1.33-fold, nominal p<0.005 in the microarray analysis). *Oxt* encodes the oxytocin protein important in maternal physiology, the abnormal expression of which has been implicated in postpartum mood disorders [21,22]. Whilst the direction of expression difference was generally consistent across the microarray and qPCR analyses for these genes, only two genes (*Cyp2g1* and *Stoml3*) were significantly differentially-expressed according to qPCR (U=34.0, one-tailed p=0.014 and t[15.3]=-1.83, one-tailed p=0.04 respectively)(**Figure 1**); for the remaining four genes, p>0.075. We next tested whether *Cyp2g1* and *Stoml3* expression was sensitive to the co-administration of vehicle or ziprasidone (0.3mg/kg or 1.0mg/kg) in 667-Coumate treated animals; the expression of neither of these genes was affected: *Cyp2g1* (F[2,28]=0.58, p=0.57) and *Stoml3* (F[2,28]=2.83, p=0.08)(**Figure 2**).

## Discussion

Although our vehicle and 667-Coumate treated groups differed substantially both in terms of their behaviour and with respect to a peripheral endocrine marker of STS inhibition (DHEAS:DHEA ratio), and the microarray experiment was performed with standard quality control procedures, we identified surprisingly few gene expression differences between the groups; those changes which we did identify by microarray were relatively small in magnitude and several were not significantly different by quantitative PCR. These data indicate that, at least at the timepoint we assayed, large brain gene expression differences do not substantially contribute towards abnormal maternal behaviour in the STS inhibition mouse model, and suggest that the behavioural differences are associated with another underlying biological mechanism e.g. the aggregate effect of small expression changes across many genes. This idea is consistent with our previous observations of: a) few large, statistically-significant, gene expression differences between whole brain samples from male mice lacking the *Sts* gene and wildtype animals [23] despite considerable between-group behavioural differences [6, 7, 8, 24, 25, 26] and b) evidence that small brain expression changes (<1.5-fold) detectable between 667-Coumate and vehicle-treated mice by qPCR, but not detectable by the present microarray study, might be associated with postpartum behavioural phenotypes [13]. We did not identify any overlap between genes significantly differentially expressed in the current study, and those whose expression was altered in whole brain from *Sts*-deficient male mice [23], and nor was there any overlap with genes whose skin expression was altered in male patients with steroid sulfatase deficiency [27]; hence, the genetic mechanisms associated with acute STS inhibition in females and constitutive STS deficiency in males may be largely dissociable.

We did observe robust upregulation of the *Cyp2g1* and *Stoml3* genes (~3-fold and 1.5-fold respectively) in 667-Coumate treated whole brain. The expression of both genes is restricted to the olfactory system in mice [28,29]. CYP2G1 is a major P450 enzyme in the olfactory mucosa of rodents, and, whilst its absence in homozygous knockout mice does not appear to impair olfaction, it does result in altered steroid hormone metabolism and metabolic activation of coumarin [28]. Increased expression of *Cyp2g1* following administration of 667-Coumate, a tricyclic coumarin sulfamate [30], is perhaps, therefore, unsurprising, and further evidence that the drug is influencing neurophysiology. In man, functional *Cyp2g1* orthologues appear to be rare [31]. Whilst the biological roles of STOML3 remain to be fully clarified, there is some evidence that the protein mediates mechanosensory processes [32]. The elevated expression of *Cyp2g1* and *Stoml3* genes in 667-Coumate-treated mouse brain could not be reduced through antipsychotic (ziprasidone) adminstration, indicating that these genes and their asociated proteins are unlikely to play a large role in mediating the rescue effect of ziprasidone on aspects of maternal behaviour.

Pathway analysis incorporating all nominally-significant microarray hits (including those with low differential expression) suggested that 667-Coumate may perturb olfactory transduction processes and that this perturbation may mediate drug-induced effects on postpartum maternal behaviour in the mouse; potentially, the increased prevalence of postpartum mood disorder in STS deficient women may be partially attributable to abnormalities within the olfactory system and/or its links to the limbic system. This possibility is feasible in light of the clinical observation that therapeutic 667-Coumate administration can elicit taste disturbances in female patients [33], and is consistent with an extensive literature on the importance of olfactory (and associated limbic) function in mammalian mothers [34], with evidence that several steroid sulfates act as ligands within the mouse accessory olfactory system [35], with the expression of multiple olfactory receptors within human brain tissue [36], and with recent findings that olfactory processes are perturbed in both a genetic mouse model of abnormal maternal behaviour [37] and multiple mood disorders [38]. With regard to specific candidate genes of interest, both [37] and the present study highlighted *Olfr59* expression as being significantly upregulated (~1.4-fold, p<0.05) in hypothalamus and whole brain respectively in mothers with maternal behavioural abnormalities; this gene has no clear human orthologue. Potentially, olfactory abnormalities could predispose to postpartum mood problems via impairing mother-offspring bonding and social judgment, through increasing sensory anhedonia, or through increasing levels of stress [38,39]. Additionally, it is important to recognise that olfactory abnormalities are likely to be only one of many biological mechanisms contributing to altered maternal behaviour.

Our study is limited in two main ways. First, we examined gene expression across the whole brain. Whilst this strategy is useful for capturing widespread expression changes and is sensible given our lack of knowledge about the underlying neuroanatomy of postpartum mood disorders, it is unlikely to detect gene expression changes between groups that are regionally-specific. Second, microarray technology, whilst cheap and easily implementable, is relatively insensitive to small gene expression changes, and has low resolution with regard to determining differentially expressed splice variants [40]. Hence, future between-group comparisons might use a more specific, and more sensitive, technique such as RNA sequencing to assay gene expression in selected brain regions of importance to maternal behaviour and mood regulation, and whose chemistry is known to be altered by STS deficiency e.g. hippocampus.
